# Feasibility of connecting regional research programs to national multisite trials emanating from the CTSA Trial Innovation Network

**DOI:** 10.1017/cts.2019.437

**Published:** 2020-01-24

**Authors:** Laurie Hassell, Charlie Gregor, Ann Melvin, Christopher Goss, Robert H. Coker, Cindi Laukes, Sandra Albritton, Jeannine Brant, Paul Amoroso, Nichole Whitener, Katherine R. Tuttle

**Affiliations:** 1 Institute of Translational Health Sciences, Seattle, WA, USA; 2 Seattle Children’s, Seattle, WA, USA; 3 Department of Medicine, University of Washington, Seattle, WA, USA; 4 Institute of Arctic Biology, University of Alaska, Fairbanks, AK, USA; 5 Neural Injury Center, University of Montana, Missoula, MT, USA; 6 Kootenai Health, Coeur d’Alene, ID, USA; 7 Billings Clinic, Billings, MT, USA; 8 MultiCare Health System, Tacoma, WA, USA; 9 Saint Alphonsus Health System, Boise, ID, USA; 10 Providence Health Care, Spokane, WA, USA

**Keywords:** Translational research, clinical research network, clinician-engaged research, multisite clinical trials, community-academic partnership

## Abstract

A collaborative research model was developed and tested to enable regional healthcare systems to join multisite clinical trials emanating from the Clinical and Translational Science Award (CTSA) Trial Innovation Network (TIN) by the Institute of Translational Health Sciences at the University of Washington and the Northwest Participant and Clinical Interactions (NW PCI) Network. The NW PCI is a collaborative group of regional research programs located at medical centers, healthcare systems, and universities across Washington, Wyoming, Alaska, Montana, and Idaho. This article describes the purpose, development, barriers, and initial experience with feasibility assessment for TIN-supported studies in the NW PCI. The tools and processes of the NW PCI Network were adapted to enable network sites to assess studies for clinical relevance and feasibility. Seven of seventeen TIN-supported studies were reviewed for consideration; three of which resulted in successful completion of study documentation for site selection by NW PCI sites. The NW PCI/TIN model can be adapted by other CTSAs to increase involvement of regional research programs in national multisite clinical research studies. Barriers to expanding TIN-supported trials to regional networks include short timelines for study document submissions, insufficient site reimbursement rates, and non-feasible study designs.

## Introduction

Translation of scientific discoveries into practice is vital to ensure better health for patients. Most patients receive care at regional or community-based medical centers [1], and yet research, especially federally funded research, is more commonly conducted in university settings. This disconnect between where patients receive care and where research is conducted limits opportunities for patients to participate in studies funded by their tax dollars and may contribute to a gap between research discovery and patient benefit [2,3] and low rates of adoption of evidence-based care for chronic conditions [4].

Substantial barriers exist for regional or community-based healthcare organizations to conduct research [5–7]. Patient care priorities may not align with research opportunities. Study protocols may not be feasible at the point of care, disrupt clinical operations, and require specialized staff, equipment, or facilities. Regional healthcare providers frequently have limited or no dedicated time to conduct research and often operate under institutional productivity models that do not support dedicated research time. These challenges are compounded by limited extramural funding that can be inadequate for administrative and investigative requirements needed for research.

The Clinical and Translational Science Award (CTSA) Program at the National Institutes of Health established the Trial Innovation Network (TIN) to address critical barriers and accelerate translation of health discoveries into practice [8]. The TIN creates an opportunity for CTSA hubs, like the Institute of Translational Health Sciences (ITHS), a CTSA-funded institute at the University of Washington [9] to establish connections between national multisite clinical studies and regional networks, such as the Northwest Participant and Clinical Interactions (NW PCI) Network. The NW PCI Network is comprised of 14 clinical and translational research centers, affiliated with medical centers, healthcare systems, and universities in Washington, Wyoming, Alaska, Montana, and Idaho (WWAMI). The governance and infrastructure of the NW PCI Network have previously been described [10].

Together, the ITHS and the NW PCI Network possess extensive expertise with funding mechanisms, multisite clinical studies, and a production model for conducting clinical and translational research across the WWAMI region. The aim of this study was to describe how the NW PCI/TIN model has been applied to studies that have come through the TIN [10] and the early experience connecting these trials with regional research centers and investigators.

## Methods

### Development

Leveraging infrastructure and expertise at the ITHS and NW PCI sites and aligning processes to facilitate research partnerships with the maturing TIN program, the NW PCI/TIN model is a multifaceted approach for WWAMI regional participation. Development of the NW PCI/TIN model included: (1) establishing criteria to determine feasibility of study protocols at sites; (2) revising review processes to assess relevance to regional clinical settings; (3) adapting ITHS TIN Hub and NW PCI tools and processes [10] to disseminate study opportunities and assess site-specific feasibility; and (4) collaborating with investigators interested in including regional network sites in their research.

NW PCI sites have existing infrastructure to support and experience to conduct a spectrum of clinical and translational research, including multisite industry, foundation and federally sponsored trials, research compliance programs, trained and experienced clinical investigators and research staff (e.g., research coordinators), and electronic health records (EHR) [10]. The NW PCI Coordinating Center at the ITHS supports investigators seeking partnerships with NW PCI sites by providing initial assessment of studies, advising on alignment with NW PCI priorities, facilitating partnerships with interested NW PCI research teams, and assisting with the development of funding proposals [10]. The ITHS Hub Liaison Team disseminates TIN-supported study opportunities, identifies investigators, assists with cohort assessments and site surveys, and supports research teams through study start-up.

Previously established NW PCI assessment procedures relied on consultations with investigators by the NW PCI Coordinating Center and review of key study information by the NW PCI Steering Committee [10]. The NW PCI Steering Committee, which meets monthly via video conference, recognized the need for regional clinician input and determined that their review was not necessary if TIN-supported studies were reviewed and approved by clinical investigators from the ITHS and NW PCI sites familiar with regional standard of care practices and clinical operations. To facilitate clinical reviews, the NW PCI Steering Committee and Coordinating Center and ITHS Hub Liaison Team refined existing assessments to define feasibility criteria based on study information typically provided by TIN coordinating centers (e.g., study synopses, clinical equipoise, draft budgets, deadlines for submission, and information about research participants) (Table 1). Clinicians affiliated with NW PCI sites and the ITHS with expertise across medical specialties (e.g., nephrology, pulmonology, infectious disease, cardiology, and internal medicine) and representing diverse clinical settings reviewed TIN-supported studies for the NW PCI Network.

**Table 1.** Study feasibility assessment criteria

Study element	Importance for feasibility assessment
Study and/or protocol synopsis	Sufficient detail is necessary to determine the research activity sites are likely to perform, and what resources and qualified personnel are needed
Site budget	Draft budgets are acceptable, but sufficient detail must be included to indicate research expenses at the sites will be covered
Research question, impact, and importance of the study	Studies must align with clinical priorities at network sites
Cohort phenotype, total number of patients, and expected recruitment rate	Inclusion and exclusion criteria, ICD-10 codes, or other descriptions are necessary to determine if sites have sufficient patients and whether recruitment can be accomplished
Deadlines for submission of study documentation	Data analysts, providers, and other clinical personnel need sufficient time to complete activities preparatory to research (e.g., electronic health record data extrapolation, completion of site surveys)
Likelihood of site selection	Time to provide study feedback and documentation is not funded, so sites must justify this investment and have a reasonable likelihood of being selected for a study
Likelihood of funding	Sites with limited capacity for research are not typically resourced to absorb costs associated with unfunded studies, so priority is given to studies with or having a higher likelihood of funding (e.g., grants that have received fundable scores)
The “Ask”- what does the principal investigator need from prospective sites	List of required items from sites (e.g., protocol feedback, letter of support, site survey, subcontract materials)

ICD-10, International Classification of Diseases, 10th revision.

### Application

TIN-supported study opportunities were disseminated to the CTSA TIN hub liaisons. The ITHS TIN Hub Liaison Team point of contact screened study opportunities based on the revised criteria, collaborated with liaisons at the lead investigators’ home institutions to establish submission of interest processes and documentation deadlines, compiled study materials into investigator engagement packets, and disseminated packets to clinical reviewers with expertise relevant to each study opportunity.

**Fig. 1.**
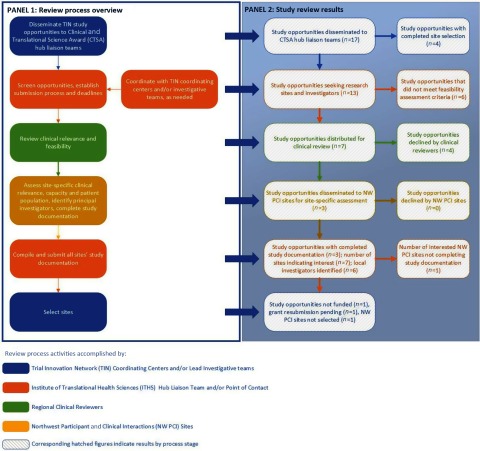
Northwest Participant and Clinical Interactions (NW PCI)/Trial Innovation Network (TIN) review process and results.

Clinicians reviewed investigator engagement packets and advised the ITHS Hub Liaison Team point of contact of their decisions to approve or decline studies. Investigator engagement packets for approved studies were disseminated via email to NW PCI site champions for site-specific assessment of clinical relevance, available capacity, patient population analysis, and identification of local investigators. Consistent with this review process, NW PCI sites assess study opportunities using several approaches: review by institutional research committees or research or clinical leadership, or less commonly, by the potential lead site investigator as is typical of university-based medical centers. Interested sites compiled the requested information and sent completed study documentation to the ITHS Hub Liaison Team point of contact, who compiled and submitted data from all sites to the TIN coordinating center assisting the lead investigative team. The ITHS Hub Liaison Team point of contact addressed questions from interested sites via email, phone, or video conference and requested additional information from the TIN coordinating centers and/or investigative teams, as needed. An overview of the study assessment, clinical relevance, and feasibility review process is described in Fig. 1 (Panel 1).

## Results

During development of the NW PCI/TIN model, between January 2018 and August 2019, 17 study opportunities were disseminated to the CTSA TIN hub liaisons, 13 of which solicited investigators and sites for multisite trials, referred to by the TIN as “expression of interest requests.” The remaining four study opportunities had already selected research sites and were utilizing other services from the TIN (e.g., centralization of IRB), precluding engagement of regional sites. Seven studies were identified by the ITHS TIN Hub Liaison Team point of contact as possible candidates for the NW PCI sites (Table 2) and distributed for clinical review. Six studies did not meet feasibility assessment criteria and were not distributed for NW PCI clinician review because target populations (i.e., pediatric and US Veterans) were served by non-NW PCI affiliated institutions (*n* = 4) and study timelines were too short (*n* = 2). Three studies were approved by clinical reviewers and disseminated to NW PCI site champions for site-specific review, resulting in seven interested NW PCI sites, six of which completed study documentation and identified a total of six local investigators (Table 2). Four studies were declined after clinical review because an intervention was already considered standard of care (*n* = 1), conditions were unlikely to be encountered in most regional sites (*n* = 2), and a protocol was unlikely to be feasible in regional clinical settings (*n* = 1). Of the three studies in which NW PCI sites completed documentation, one was not funded, a grant resubmission is pending on another, and the lead investigative team did not select NW PCI sites in the third. Fig. 1 provides an overview of the clinical relevance and feasibility review process (Panel 1) aligned with the results of the study review at each stage (Panel 2).

**Table 2.** Summary of NW PCI/TIN results

Study/EOI	NW PCI sites	Outcomes	Study/application status
New diagnostic testing schema for angina	Providence Health Care, Spokane, WA MultiCare Health System, Tacoma, WA Kootenai Health, Coeur d’Alene, ID	Site principal investigators (Providence and MultiCare) Written feedback on protocol design and budget (Providence and MultiCare) Cohort assessment (Providence and MultiCare) Declined to participate (Kootenai)	Not funded
Clinical trial of a drug treatment for heart failure	Study declined by clinician reviewers	Intervention was already considered standard of care, precluding randomization	Unknown
Test of a personal device technology for arrhythmia monitoring	MultiCare Health System, Tacoma, WA Saint Alphonsus Health System, Boise, ID	Site principal investigators Written feedback on protocol design and budget Cohort assessment	Grant resubmission pending
Study of patient outcomes after ICD generator exchange	Providence Health Care, Spokane, WA MultiCare Health System, Tacoma, WA	Site principal investigators Written feedback on protocol design and budget Cohort assessment	NW PCI sites not selected
Comparative effectiveness study for rare heart condition	Study declined by clinician reviewers	Rare condition was unlikely to be encountered at most sites	Unknown
Study evaluating brain oxygenation during cardiac arrest	Study declined by clinician reviewers	Feasibility, including budget concerns	Unknown
Feasibility study of visual and auditory recall during cardiac arrest	Study declined by clinician reviewers	Condition was unlikely to be encountered at most sites	Unknown

NW PCI/TIN, Northwest Participant and Clinical Interaction/Trial Innovation Network; EOI, expression of interest.

All studies submitted to the NW PCI for clinical review were in the therapeutic area of cardiovascular disease and included a new diagnostic testing schema for angina, a clinical trial of a drug treatment for heart failure, a test of a personal device technology for arrhythmia monitoring, a study of patient outcomes after implantable cardioverter defibrillator (ICD) generator exchange, a comparative effectiveness study for a rare heart condition, a study evaluating brain oxygenation during cardiac arrest, and a feasibility study of visual and auditory recall during cardiac arrest. For the study of a new diagnostic testing schema for angina, two NW PCI sites (Providence Health Care, Spokane, Washington and MultiCare Health System, Tacoma, Washington) identified local site champions, provided written feedback on the protocol design and budget, and submitted an EHR cohort assessment. A third site (Kootenai Health, Coeur d’Alene, Idaho) expressed interest in the study, but did not submit study documentation by the deadline. This first study provided a paradigm for development and implementation processes for NW PCI review, dissemination of study information, and collection of required study documentation from potential NW PCI sites.

The review of a clinical trial for drug treatment of heart failure concluded that the proposed intervention was largely considered standard of care among NW PCI health systems and was declined because sites would be unlikely or unwilling to randomize participants.

**Fig. 2.**
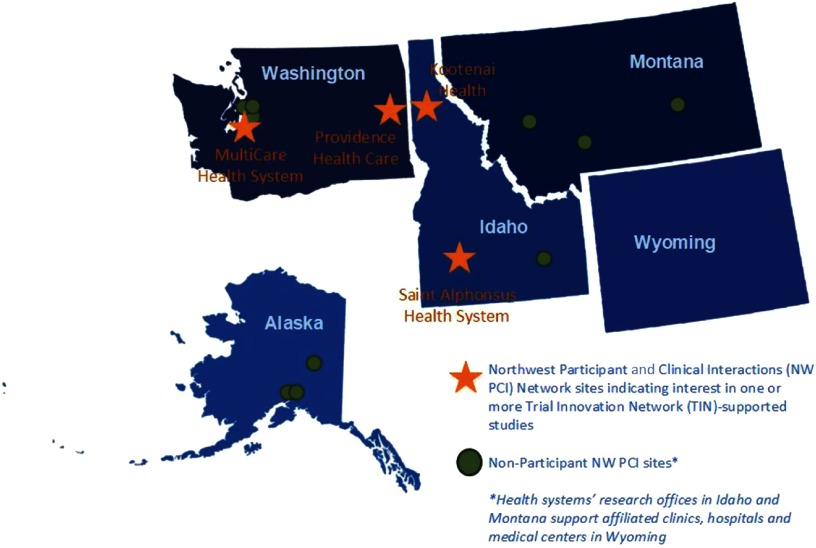
Northwest Participant and Clinical Interactions (NW PCI)/Trial Innovation Network (TIN) participants and non-participant sites.

NW PCI sites identified site principal investigators and submitted required study documentation for the third study opportunity, a test of a personal device technology for arrhythmia monitoring (MultiCare Health System and Saint Alphonsus Health System, Boise, Idaho), and fourth study assessing patient outcomes after ICD generator exchange (Providence Health Care and MultiCare Health System). Fig. 2 indicates the location of all NW PCI sites and highlights sites participating in one or more of the TIN-supported studies (Table 2). Reviewers declined a comparative effectiveness study of treatment for a rare heart condition, and a pilot study examining conscious awareness in deep hypothermic circulatory arrest patients because the studies involved conditions that are not commonly encountered at regional sites. Infeasibility of the research protocol, including concerns about insufficient funding to support sites’ research activities, was the rationale for declination of a feasibility study of visual and auditory recall during cardiac arrest.

Initial feasibility testing of the NW PCI/TIN model with these first seven studies informed processes for all studies in which investigators seek partnerships with NW PCI sites. Feedback from NW PCI sites, both interested and not interested in particular study opportunities, was used to refine the study feasibility assessment criteria described in Table 1. The NW PCI Steering Committee revised the overall process for study reviews, incorporated the NW PCI/TIN enhanced assessment criteria (Table 1), implemented a clinician review process, and discontinued secondary review of study information. New online content was uploaded to the ITHS website (e.g., revised assessment criteria, recommended timeline) [11]. A web form for study submission is in development to facilitate more efficient onboarding of all NW PCI study opportunities.

## Discussion

Feasibility testing of the NW PCI/TIN model demonstrated how a successful, collaborative framework can enable regional research programs to participate in TIN-supported multisite clinical trials [10]. NW PCI sites were able to complete feasibility assessments and submit study documentation for site selection consideration in 3 of the 17 TIN-supported studies. The process screened out the other 14 studies. Review of studies by clinicians familiar with regional standards of care, clinical operations, and research policies was central to the development of the NW PCI/TIN model. Implementation of study feasibility assessment criteria and clinical review for NW PCI studies streamlined NW PCI Coordinating Center processes and eliminated the need for secondary reviews by NW PCI Steering Committee members.

The incorporation of study feasibility assessment criteria from regional sites into study designs is an important consideration for investigators to enable greater clinical relevance to regional healthcare settings and is essential for successful collaboration on multisite trials. Adaptation of feasibility assessment criteria for the NW PCI/TIN feasibility study is an early model for the TIN experience. Because healthcare organizations have varying priorities and capacities and serve diverse local and regional patient populations, customized criteria should be established collaboratively by CTSAs and their research partners. Long-term partnerships between the ITHS and NW PCI sites formed a foundation for the NW PCI/TIN model. Relationships must be built between CTSAs and healthcare organizations outside of their home institutions to establish customized feasibility criteria and ensure feasible study designs for regional collaborations to succeed.

CTSAs are positioned nationally through the local TIN hub liaisons to identify common feasibility assessment themes across regional partners to improve the overall feasibility of all TIN-supported studies, including those in which site selection is complete. Tracking and incorporation of common feasibility assessment themes may facilitate collaboration across CTSA hubs and their partners, potentially streamlining site selection, particularly for studies requiring many research sites, or involving unique populations or rare diseases.

Realistic timelines to identify local investigators, to submit study documentation, and to provide feedback are a critical consideration that directly impacts NW PCI site participation in TIN-supported studies. While study start-up time frames for multisite trials in regional NW PCI sites are comparable with those expected at university-based medical centers, pre-award time frames must often accommodate site research review schedules. The process to identify site principal investigators, and complete review, candidate population assessments, and study documentation typically requires at least 8 weeks. Participation in TIN-supported studies by regional research programs is likely to meaningfully increase with longer submission deadlines or reduced burden of submission (i.e., site selection surveys versus EHR cohort assessments).

The NW PCI/TIN model was tested in seven studies for which the study design was largely determined prior to soliciting feedback from regional NW PCI sites. While this approach identified studies that were feasible in regional healthcare settings, the research questions, methodologies, and budgets were determined prior to solicitation of input from regional sites. The rationale for declination by clinicians of three studies, for example, inability to randomize an intervention that was largely standard of care or without an accessible population, underscores the importance of early review by regional stakeholders. Regional healthcare organizations have limited capacity for new study development at very early stages (e.g., research question and aims development in the pre-award phase), which is a barrier to participation. Facilitating access to funded post-award studies, or those late in development or with fundable grant scores, would increase the likelihood of regional site involvement. If greater opportunity is provided for regional stakeholders to participate in study development, CTSA hubs will be uniquely positioned to leverage academic and regional expertise to improve the overall quality of grant applications.

Site selection was completed for some studies prior to dissemination to the ITHS Hub Liaison Team, precluding involvement with and assessment of relevance and feasibility within NW PCI sites. CTSAs should consider how TIN-supported studies and investigators affiliated with their hubs may benefit from regional participation, prior to site selection. Many regional research centers have excellent capacity and operations, as well as access to underserved populations for research. Their participation earlier in the study development process without overburdening sites with pre-award study opportunities could be accomplished by convening standing panels or pools of *ad hoc* specialists affiliated with CTSAs’ regional partners to evaluate study ideas. Alignment with clinical priorities and feasibility in healthcare settings outside of the CTSA Consortium are important contributions that regional programs bring to research partnerships. Reviewers can facilitate development of research partnerships between CTSAs and regional partners for efficient site selection and allow research access for patients closer to home.

The NW PCI sites with their extensive experience and capacity for research served as ideal partners for the NW PCI/TIN model. NW PCI sites participating in this feasibility study are not contractually affiliated with the ITHS (e.g., members of CTSA-allied health systems, memoranda of understanding), which may contribute to the generalizability of the model to other CTSAs seeking partnerships with unaffiliated regional healthcare organizations. However, adaptation of the NW PCI/TIN model is dependent on partnerships, which require time and ongoing investment by CTSAs, so implementation will require CTSAs investing in regional relationships. Further assessment of partnerships between NW PCI sites and TIN-supported investigators is limited because the three studies with potential NW PCI sites are either on-hold pending funding, were not funded, or did not select a NW PCI site. Although the NW PCI/TIN model involved seven cardiology studies, involvement of clinicians across different therapeutic areas is necessary to review a broad range of TIN-supported studies.

Integration of patient input early in study development is of critical importance for the success of multisite clinical trials. Patients’ perspectives inform development of research questions to address their health priorities, research protocols to promote recruitment and retention, and recruitment plans to increase enrollment of relevant patient populations. The ITHS plans to leverage learnings from current community- and patient-engaged research studies (e.g., NIH “moonshot” and other precision medicine initiatives) to adapt the NW PCI/TIN model for earlier involvement of patient stakeholders.

In conclusion, the ITHS and the NW PCI Network have developed a model that brings together national TIN-supported investigators and regional research programs with experience and infrastructure to support multisite clinical studies. The NW PCI/TIN model leverages the convening functions of the CTSAs to bring together investigators and organizations to facilitate development of research partnerships. By creating a bridge between them, the NW PCI/TIN model facilitates conduct of research in diverse clinical settings where most patients receive their care and enable local access for patients to high-quality research studies supported by their tax dollars.
